# A health economic model for evaluating a vaccine for the prevention of herpes zoster and post-herpetic neuralgia in the UK

**DOI:** 10.1186/1478-7547-8-7

**Published:** 2010-04-30

**Authors:** Lee Moore, Vanessa Remy, Monique Martin, Maud Beillat, Alistair McGuire

**Affiliations:** 1 i3 Innovus, Uxbridge, UK; 2 Sanofi Pasteur MSD, Lyon, France; 3 London School of Economics, London, UK

## Abstract

**Background:**

A live-attenuated vaccine aimed at preventing herpes zoster (HZ) and its main complication, post-herpetic neuralgia (PHN) is available in Europe for immunocompetent adults aged 50 years and more. The study objective is to assess the cost effectiveness of a vaccination program for this population in the UK.

**Methods:**

A state-transition Markov model has been developed to simulate the natural history of HZ and PHN and to estimate the lifetime effects of vaccination in the UK. Several health states are defined including good health, HZ, PHN, and death. HZ and PHN health states are further divided to reflect pain severity.

**Results:**

The model predicts that a vaccination strategy for those aged over 50 years would lead to an incremental cost-effectiveness ratio of £13,077 per QALY gained from the NHS perspective, when compared to the current strategy of no vaccination. Age-group analyses show that the lowest ICERs (£10,984 and £10,275 for NHS) are observed when vaccinating people between 60-64 and 65-69 years of age. Sensitivity analyses showed that results are sensitive to the duration of vaccine protection, discount rate, utility decrements and pain severity split used.

**Conclusions:**

Using the commonly accepted threshold of £30,000 per QALY gained in the UK, most scenarios of vaccination programmes preventing HZ and PHN, including the potential use of a repeat dose, may be considered cost-effective by the NHS, especially within the 60 to 69 age-groups.

## Background

In our aging society, diseases whose prevalence rises with age are of increasing significance. The varicella zoster virus (VZV) causes chickenpox as the primary infection. Reactivation of the dormant virus in the dorsal root ganglia of individuals having had a primary infection can occur, causing herpes zoster (HZ) or "shingles". The lifetime risk of suffering from HZ is 25% [1]. HZ incidence increases sharply with age, roughly doubling in each decade past the age of 50 years [2]. Normal age-related decrease in varicella zoster-specific cell-mediated immunity is thought to account for this increased incidence of VZV reactivation. The symptoms of HZ include numbness, itching and pain during the prodromal phase (before the rash onset), followed by painful unilateral vesicular eruptions on the skin. The pain and cutaneous eruptions usually subside after 3-4 weeks [3].

In about 20% to 25% of HZ cases [4], pain can persist for months or years after the cutaneous eruption has healed. Depending on the definition used, pain occurring or persisting at least 1 or 3 months after rash onset is termed post-herpetic neuralgia (PHN). While there is no international consensus on the definition of PHN, it is increasingly commonly accepted that PHN is considered to be present if pain persists for more than 3 months or more after rash onset [5,3,6]. With age as its primary risk factor[4], the incidence of PHN is expected to increase in an ageing population. Many patients with PHN go on to develop severe physical, occupational and social disabilities as a consequence of the enduring pain [4].

A new live attenuated vaccine (Zostavax^®^) against varicella zoster virus infection is indicated in Europe for the prevention of HZ and HZ-related PHN from the age of 50. The efficacy of this new vaccine has been tested in a randomized, double-blind, placebo-controlled trial (Shingles Prevention Study, SPS) [6]. This study, involving 38,546 immunocompetent patients aged 60 years and older, reported that vaccination reduced the incidence of HZ by 51.3% and reduced the severity of pain when HZ occurred in vaccinated individuals. Progression to PHN was reduced by 66.5%, with a trend toward greater efficacy for PHN of longer duration [6]. In addition, the safety and immunogenicity of the vaccine in the 50-59 age groups has been explored in two clinical trials which showed that the vaccine was well tolerated among patients aged 50 years and older and induced an immunogenic response not inferior to that of subjects 60 and older and thereby provided an immunological bridge to vaccine efficacy demonstrated in the SPS (ref Oxman, 2005) [7,8].

In order to estimate the potential effectiveness and cost-effectiveness of a vaccination programme against HZ and PHN in the UK, we have used a cohort model that strikes a balance between real-life individual variability and the more formal structures required for modelling. The model aims to accurately represent the epidemiology of HZ and PHN. In addition it aims to quantify the lifetime health benefits and economic effects of vaccination in the UK immunocompetent population aged 50 and over.

## Methods

A Markov model was developed to compare two policies: the implementation of a vaccination policy in the UK, where a predetermined vaccine coverage rate is achieved for each age group at the start of the model, and the current policy in the UK of no vaccination. Under these two policies, the following outcomes were compared for the lifetime: HZ cases and PHN cases, quality-adjusted life-years (QALYs) and total costs. These outcomes resulted in the calculations of incremental cost-effectiveness ratios (ICERs), including cost per QALY gained, cost per HZ case avoided, and cost per PHN case avoided, and the number needed to vaccinate (NNV) to prevent one occurrence of HZ or PHN. Both a NHS (National Health Service) perspective and a societal perspective were considered. The NHS perspective includes all health care related expenses, as the national health care system is tax-funded and universally accessible. The societal perspective includes in addition to healthcare expenses, productivity costs due to time off work, but no co-payments.

### Model features

**Figure 1:**
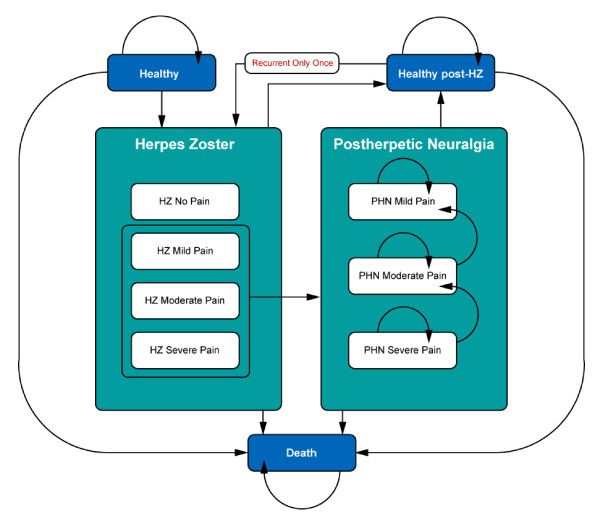
**Model Health States**.

This model utilises a Markov process to simulate the lifetime incidence and consequences of HZ among a population aged 50 and over, corresponding to the therapeutic indication of the HZ vaccine Zostavax^®^. The model was developed in *Microsoft Excel 2003*. The population is analysed as separate 5 year age cohorts (*i.e*. the 50-54 year old population is first analysed over its lifetime, then the 55-59 year old population, *etc*.). The state-transition Markov model describes several health states including healthy (*i.e*. no HZ symptoms), HZ, PHN, and death. HZ and PHN health states are further divided into different pain severity levels. Recurrent HZ and the subsequent neuropathic pain are also allowed states, but are constrained to a one-time only recurrent episode. Figure 1 shows the potential health states, with arrows indicating possible transitions between states. The model runs in Markov cycles of one month to represent the average duration of an episode of HZ. Within each 1 month cycle, the cohort members may remain in their current health state or transit to one of the allowable states. Transitions are governed by a matrix of probability values. With each successive cycle, an increasing proportion of the cohort moves through the HZ and PHN states and eventually to death. The model runs through sufficient cycles until the entire cohort has died.

Movement from healthy to "HZ" is based on monthly age-specific HZ incidence rates. Individuals are allocated to one of the four HZ pain severity levels (no, mild, moderate and severe pain). In the following month, a proportion of these HZ patients will develop PHN. The literature indicates that patients have a higher risk of developing PHN if they experience severe pain during HZ. To reflect the increased (decreased) odds of developing PHN by patients suffering from a severe (mild) HZ pain episode relative to moderate HZ pain information from the literature was used by applying odds ratios of 2.39 for severe pain and 0.88 for mild pain [9]. Hence, patients with severe (mild) pain are 2.39 (0.88) times more (less) likely to develop PHN than patients with moderate level of pain.

New PHN patients were distributed over three initial pain severity levels (mild, moderate, or severe) determined at diagnosis. It was assumed that the PHN-related pain diminishes over time and eventually ceases. Therefore, an individual can either remain in the same state or transition to a less severe PHN pain state each month. All must go through the mild PHN pain state to attain a healthy (post-zoster) state. Consequently, the average duration of PHN depends on the initial PHN pain split of the population (as it will take longer for 'severe' patients to return to health than for 'mild' patients) and on the transition probabilities used between PHN pain states and the post-HZ healthy state. It was therefore essential to calibrate these transition probabilities to reflect the average duration of PHN from the literature. The model assumed that the transition rate from severe to moderate PHN, moderate to mild, and mild to healthy (post-zoster) were identical. This constant rate differed by age group as older patients experience longer duration of pain [6].

This model has a special state for recurrent HZ as this is an extremely rare condition. Cunningham [10] reported a 1%-5% lifetime risk of recurrent zoster. The mid-point from the Cunningham data, 3%, was used to determine the incidence of recurrent HZ by applying it to the average life expectancy for individuals having experienced a first zoster episode. Characteristics of a recurrent HZ patient (duration, initial pain split, subsequent PHN) was assumed to be identical to the first-time experience.

Age-specific mortality rates were applied to each health state to determine the monthly probability of transitioning to "Death".

### Model Inputs

#### Input Sources

Inputs used to populate the model were derived from several sources presented in Tables 1 and 2. Our model was mainly informed based on a recent retrospective UK database analysis [11]. This provided the model with recent epidemiological and healthcare resource use information with the exception of hospitalisations and productivity losses. Ultimately, additional data from the literature were used to populate selected input parameters, such as hospitalisation rates [12], utility decrements [13], pain split and PHN duration [6]. Moreover, two parameters (days of work lost due to HZ and PHN and probable vaccine administration characteristics) have been made up on expert opinions due to the lack of data in these matters.

**Table 1:** Input Data: Epidemiology, Vaccine Characteristics and Utilities

	Base case		Deterministic sensitivity analysis	
	HZ	PHN	HZ	PHN
**Epidemiology**				
Annual HZ Incidence (per 1,000 people)/PHN Proportion per HZ case	*Gauthier 2008 *[11]		*Edmunds 2001 *[12]	
Age 50 - 54	3.34	10.30%	4.61	-
Age 55 - 59	4.08	13.70%	5.21	-
Age 60 - 64	4.90	15.70%	5.92	-
Age 65 - 69	5.96	18.70%	6.70	-
Age 70 - 74	6.34	22.50%	7.53	-
Age 75 - 79	7.09	26.60%	8.42	-
Age 80 - 84	7.29	28.90%	9.37	-
Age 85+	6.22	25.90%	11.58	-
Mean Duration (in months)	*SPS Trial 2005 *[6]		*Gauthier 2008 *[11]	
Age ≤ 69	1.0	10.3	-	10.9
Age ≥ 70	1.0	12.9	-	11.0
Annual HZ Mortality Rate	*Assumption*		*Edmunds 2001 *[12]	
Age 50 - 54	0%	-	0.0009%	-
Age 55 - 59	0%	-	0.0009%	-
Age 60 - 64	0%	-	0.0027%	-
Age 65 - 69	0%	-	0.0027%	-
Age 70 - 74	0%	-	0.0035%	-
Age 75 - 79	0%	-	0.0092%	-
Age 80 - 84	0%	-	0.0487%	-
Age 85+	0%	-	0.2018%	-
Gender split	*Gauthier 2008 *[11]			
% female	61%	65%		
Pain severity split at diagnosis	*SPS Trial 2005 *[6]		*Gauthier 2008 *[11]	
Age ≤ 69				
No pain	27%	-	65%	-
Mild pain	41%	42%	24%	47%
Moderate pain	18%	9%	4%	42%
Severe pain	14%	49%	8%	11%
Age ≥ 70				
No pain	26%	-	45%	-
Mild pain	32%	17%	41%	34%
Moderate pain	23%	16%	5%	54%
Severe pain	19%	67%	9%	12%
Recurrent annual HZ Incidence				
Age 50 - 54	0.10%	-		
Age 55 - 59	0.12%	-		
Age 60 - 64	0.15%	-		
Age 65 - 69	0.18%	-		
Age 70 - 74	0.23%	-		
Age 75 - 79	0.31%	-		
Age 80 - 84	0.42%	-		
Age 85 - 89	0.58%	-		
Age 90 - 94	0.83%	-		
Age 95 - 99	1.15%	-		
Age 100+	1.40%	-		
**Quality of Life**				
Utility Decrements	*Oster 2005 *[13]		*SPS Trial - Pellissier 2007 *[15]/Bala 1998 [29]	
No pain	0.00	-	0.14/0.00	-
Mild pain	0.31	0.31	0.23/0.27	0.23/0.27
Moderate pain	0.42	0.42	0.32/0.40	0.32/0.40
Severe pain	0.75	0.75	0.45/0.53	0.45/0.53
**Vaccine characteristics**	*SPS Trial 2005 *[6]			
Efficacy: total % reduction in cases (PHN direct effect)				
Age ≤ 69	63.9%	66.7% (4.8%)		
Age ≥ 70	37.6%	66.8% (30.0%*)		
Efficacy: number of months reduction of PHN pain				
Age ≤ 69	-	-2.2		
Age ≥ 70	-	-3.3		

*Calculated based on the midpoint lifetime risk of HZ recurrence from Cunningham *et al*. [10] and UK life expectancy rates from national statistics [19].

**Table 2:** Input Data: Healthcare Resource Use (HCRU) and Costs in the UK

	HZ	PHN
**GP visits monthly costs**	*Gauthier 2008 *[11]	
No pain	£18.98	-
Mild pain	£43.66	£26.18
Moderate pain	£54.49	£26.91
Severe pain	£64.60	£25.72
**Specialist visits monthly costs**	*Gauthier 2008 *[11]	
No pain	£4.03	-
Mild pain	£4.53	£0.81
Moderate pain	£6.24	£2.04
Severe pain	£6.89	£2.81
**Medication monthly costs**	*Gauthier 2008 *[11]	
No pain	£37.14	-
Mild pain	£43.37	£10.02
Moderate pain	£46.17	£14.06
Severe pain	£53.40	£23.06
**Hospitalisation costs**		
Hospitalisation rate (painful cases only)	*Edmunds 2001 *[12]	
Age 50 - 54	0.70%	
Age 55 - 59	0.80%	
Age 60 - 64	1.10%	
Age 65 - 69	1.70%	
Age 70 - 74	2.30%	
Age 75 - 79	3.00%	
Age 80 - 84	5.20%	
Age 85+	5.90%	
Length of hospital stay	*Hospital Episode Statistics 2004-2005 *[17]	
Duration in days	9.9	11.2
Unit Costs	*NHS Reference costs 2005-2006 *[18]	
Daily cost of inpatient stay	£117.00	
**Productivity Costs**		
Days off work	*Assumption*	
No Pain	8.8	-
Mild Pain	9.6	8.8
Moderate Pain	12.3	31.5
Severe Pain	21.0	70.5
Unit cost	*ASHE 2005 *[20]*(Assuming 7.5 hour work day)*	
Cost per day lost	£93.75	
**Vaccination costs**		
Unit cost	*Assumption*	
One dose of vaccine	£95.00	
Administration cost	*PSSRU 2005 *[22]/*Assumption*	
50% GP - 50% Nurse	£10.40	

#### Epidemiology of HZ and PHN

Epidemiological inputs are provided in Table 1. HZ incidence rates for 2000-2006 were used from the GPRD analysis, described above, based on a sample of 27,225 patients from the UK [11]. The SPS trial provided data by patient group for the duration of pain in HZ [6]. These confirmed that most patients experience a 30-day duration of pain, as reported in the literature [9,14].

The monthly-cycle model structure requires utilisation of PHN data based on a 1-month definition. Where only PHN data was available using a 3-month definition, appropriate input values using a 1-month definition were calibrated in order to match the available 3-month data following 3 months of the Markov process. The PHN proportion was estimated from the GPRD database [11]. It was assumed that only HZ states associated with pain could result in PHN, as it is unlikely that PHN will ensue if no pain is present during the HZ eruption. Therefore, PHN proportion was adjusted in the model in order to be derived only from painful HZ states, while still retaining the overall PHN proportion level obtained from the database analysis [11].

An important aspect of the model was the split between the different pain states for HZ, as these are linked to PHN probability and treatment costs. SPS data [6,15] were used for HZ and initial PHN pain severity. This provided information on HZ severity levels by means of a special questionnaire specifically developed to assess HZ and PHN associated pain (the Zoster Brief Pain Inventory, or ZBPI [16]). SPS results were also selected for PHN duration, as pain severity and duration are linked in the model and therefore should be retrieved from the same source.

The GPRD analysis found that women accounted for 61% of all HZ cases and 65% of all PHN cases [11]. This affected several aspects of the model, including the utility decrements, as women experience systematically lower age-specific utility values than men, and productivity costs, since women have lower employment rates among the applicable age groups in the UK.

Information from the GPRD analysis indicated no mortality directly linked to HZ or PHN. This was confirmed by the lack of literature on the subject, however rates reported by Edmunds *et al*. 2001 were tested in sensitivity analyses.

#### Resource utilisation and costs

All costs in Table 2 are presented in 2006 UK £s. The model assumes that patients are diagnosed and treated by GPs. Referrals to specialists are subsequent to at least one GP visit. Mean monthly health care costs per HZ and PHN case were obtained from the GPRD analysis by severity. The analysis found no significant relationship between age and health care costs, thus values were applied in the model only for severity. As expected, pain severity increases costs [11].

Due to limitations of GPRD regarding inpatient data, the literature was reviewed as a supplement to select an appropriate approximation for HZ and PHN hospitalisation rates. Information from the 2004-2005 Hospital Episode statistics [17] (HES) for the number of HZ and PHN hospitalisations could be closely replicated by applying the Edmunds [12] reported hospitalisation rates (also based on the HES) to all HZ cases which experienced pain (that is, only HZ associated with mild, moderate, or severe pain) and all PHN cases. Therefore, the Edmunds hospitalisation rates [12] were used. The mean length of hospital stay was also taken from the HES [17] using the appropriate ICD-10 codes (HZ: B02 codes excluding B02.2 and PHN: B02.2). The daily cost associated with a non-elective inpatient stay due to viral illness from the NHS Reference Costs [18] was applied to calculate the total hospitalisation cost per inpatient case.

Productivity losses were based on the number of workdays lost due to disease. The data concerning the number of workdays lost were gathered from expert opinion. Experts emphasised the need for HZ patients, even with no pain, to abstain from work, as they would be infectious during the early phases of their HZ infection. UK employment data [19] and average wage values [20] were gathered to compute the average daily productivity losses, which were only included for persons aged 69 or less.

#### Vaccination characteristics

Vaccine efficacy was taken from the clinical trial SPS [6]. The efficacy data for age groups 50-59 years were assumed to be identical to ones for the 60-69 age group as this can be supported by the available immunogenicity results [7,21] providing an immunological bridge to vaccine efficacy demonstrated in the SPS [6,10]. Several effects of vaccination are included in the model, both direct and indirect (Table 1) (see Additional File 1). The vaccine has a direct effect on the number and severity of HZ and PHN cases, which differed by age. Since HZ must precede PHN, the number of PHN cases is also indirectly reduced through the reduction in the HZ cases. In addition, vaccination reduces the duration of PHN. In the model, this indirectly affects the pain severity experienced by the patient since it implies a shorter period of time spent in each painful PHN health state. This was incorporated in the model by adjusting the age-specific transition probabilities between PHN states for both vaccinated and non-vaccinated individuals, as described earlier. These adjustments reflect the vaccine efficacy in reducing the Burden of Illness (BOI is a severity-by-duration measure of the total pain), defined as primary clinical endpoint of the SPS.

In the base case, the vaccine efficacy is expected to last a lifetime. While the efficacy of the vaccine may diminish over time, the lengths of the clinical trials are currently not sufficient to accurately document this, though more reliable data may become available in the future. A waning function, currently set at 0% as described in the literature [15], has been included in the model for this purpose. Alternative assumptions examining shorter vaccine efficacy durations and including the need for a repeat dose or possible waning in vaccine efficacy were considered in the sensitivity analysis.

A vaccine coverage rate of 40% was assumed for all ages from the age of 50. The current reported coverage in the UK in the over 65 year old age group for influenza vaccine (approximately 70% [20]) or pneumococcal polysaccharide vaccine (64% [21]) was seen as the absolute maximum attainable vaccine coverage rate for the vaccine against HZ. Thus, the selected rate of 40% was perceived to be an attainable yet realistic rate for base-case.

Costs related to the administration of the vaccine were included. We assumed that in 50% of cases the vaccine would be administered by a nurse during a vaccination appointment and in 50% of cases by a GP during a routine visit. For the latter case, as patients would be vaccinated during a routine visit only the costs specific to the administration of the vaccine were included. Unit costs associated with vaccination administration were derived from the Personal Social Services Research Unit (PSSRU) [22].

#### Utilities

Age-specific utilities were used from the Health Survey for England [23]. Those are for 10-year age bands. 5-year age-specific utility values were obtained from the Canadian Health Utilities Index (HUI) Mark 3 [24] and extrapolated to the UK population (see Additional File 2). As regards to the decrement caused by pain, the model used utilities derived from Oster [13], which were obtained in a PHN population and were utilised for HZ as well, as PHN and HZ pain are both neuropathic in nature. It is expected that the utility associated with the different levels of pain severity will not vary between the various causes of neuropathic pain. In particular, a recent publication McDermott *et al*. [25] investigated the quality of life in neuropathic pain patients in five European countries reporting utility weights by severity. These weights are very similar to the values reported by Oster, indicating that the latter are appropriate to feed the model. In addition, according to expert opinion while age-specific utilities do differ between countries, the decrements due to disease may be considered similar, and thus the US Oster decrements were considered suitable for the UK analysis.

To calculate utilities, the decrements related to the pain states are applied to the age-specific utilities using an additive approach, *i.e*. pain decrements are subtracted from the age-specific utilities (see Additional File 2).

#### Population characteristics

The population size was obtained from the UK national statistics office [19] and totals 20,174,786 persons aged 50 and older as reported for mid-year 2004. Cost-effectiveness was analysed by 5-year age-groups within this population UK general mortality rates were obtained from the government actuarial department [26].

#### Discounting

The discount rate used for the base case analysis is 3.5% for costs and 3.5% for outcomes (QALYs) as recommended by NICE. There is controversy regarding whether monetary costs and health benefits should be discounted at the same rate or differentially, particularly when evaluating public health programmes such as vaccination [27,28]. It is often argued that the benefits of health promotion strategies should be discounted at a lower rate than those of costs, so as to prioritise health promotion and disease prevention over curative treatments [27]. As a result, different discount rates were tested in sensitivity analyses.

#### Sensitivity Analyses

The sensitivity of the base case ICER to alternative values of significant input parameters was explored by varying these within feasible ranges. A number of univariate sensitivity analyses were carried out in order to evaluate the effect of different parameters on the results and to identify the variables driving the results. This included variations on the discount rate, HZ incidence and mortality assumptions [12], vaccine efficacy, duration of vaccine efficacy (varied by both an assumption of limited duration of full vaccine efficacy followed by no efficacy and also by utilising a waning rate [15]) and utilisation of a vaccine repeat dose at 10 years after first dose, vaccination coverage rates by age, health care costs, vaccination costs, utility decrements [15,29], and the HZ/PHN pain split used [11]. A probabilistic sensitivity analysis (PSA) was also performed and several parameters were included. In particular, HZ vaccine efficacy was tested by varying the base efficacy using a beta distribution and by including a waning rate [15] varied uniformly. HZ mortality was also varied uniformly from the base scenario value of 0% to the age-specific values found in the Edmunds et al. publication [12]. HZ and PHN utility decrements were tested using a lognormal distribution [13]. Data gathered from the GPRD analysis, including health care resource costs and HZ incidence and PHN proportion, were varied over a normal and beta distribution respectively [11].

### Validation

Once the model had been developed, it was validated by comparison to the available clinical data. Using the clinical information from the SPS trial comparing vaccine with placebo [6], we were able to replicate exactly the number of HZ cases and PHN cases (using a 3-month definition, reflecting the primary PHN endpoint definition considered in the trial) seen in the SPS placebo arm over a period of three years (the duration of the clinical trial), 642 and 80 cases, respectively. For the vaccine arm we obtained the identical number of HZ cases reported in the trial (315 cases) [6,30]. Because there are two PHN endpoints of interest (1 and 3-months definition), the direct PHN vaccine efficacy input parameter was adjusted in the model in order to best reflect the efficacy in reducing the incidence of PHN. As a result, the PHN vaccine efficacy input parameter used in the model overestimated PHN using a 1-month definition by 0.3 percentage points (59.2% compared to the trial reported 58.9% [6,30]) and underestimated PHN using a 3-month definition by 0.3 percentage points (66.2% instead of the trial reported 66.5% [6,30]). However, as this estimation error is quite small, we were still able to replicate the number of PHN (3-month definition) cases in the vaccine arm (27 cases). Therefore all primary clinical endpoints from the SPS were successfully replicated in the model, providing confidence that the model generates a valid estimate of clinical benefit.

## Results

### Base Case

The base case results predict that the vaccination would bring benefits in the form of reduced numbers of HZ and PHN cases and QALYs gained at additional cost.

Over the lifetime of the current aged 50 and older UK population, without vaccination, it is estimated that there would be 2,307,719 cases of HZ, and either 521,616 or 477,532 cases of PHN using a 1- or 3-month definition, respectively. By comparing this to the vaccination strategy with a 40% coverage rate, there would be 22.7% fewer HZ cases, 26.6% fewer PHN cases using a 1-month definition, and 28.9% fewer PHN cases using a 3-month definition. More PHN cases are avoided when using a 3-month definition due to the shorter duration of PHN for the vaccinated population which results in more episodes resolving within 3 months. Over a lifetime, a total of 57,589 quality-adjusted life years would be gained by such vaccination strategy. The model also calculated the number needed to vaccinate in order to avoid one case of HZ and PHN (both definitions), namely 15 and 58 respectively.

The estimated costs varied according to the perspective taken either from NHS or from society. The difference in costs between the two perspectives provides information on the total productivity losses due to HZ and PHN. Over a lifetime of the current aged 50 and older UK population (no vaccination policy population), approximately £363 million or 3.9 million workdays are lost due to HZ and PHN. Accordingly, the ICERs for the societal perspective are notably lower than for the NHS perspective.

The resulting ICERs, presented in Table 3, were £13,077 per QALY gained, £1,440 per HZ avoided, £5,421 per PHN (1-month definition) avoided and £5,453 per PHN (3-month definition) avoided from the NHS perspective, and £11,417 per QALY gained, £1,258 per HZ avoided, £4,733 per PHN (1-month definition) avoided and £4,761 per PHN (3-month definition) avoided from the societal perspective.

**Table 3:** Base Case: Lifetime Results for the 50+ Age Group

Results	Lifetime horizon	TPP	Societal
ICERs	Cost per QALY	£13,077	£11,417
	Cost per HZ Case Avoided	£1,440	£1,258
	Cost per PHN Case Avoided (1 month def)	£5,421	£4,733
	Cost per PHN Case Avoided (3 month def)	£5,453	£4,761

**Figure 2:**
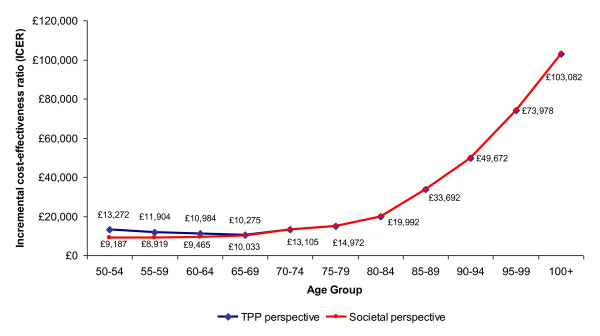
**Base Case ICERs by Age-Group**. Note: The ICERs of the two perspectives are identical after the age of 70 as there are no productivity losses after this age.

Separate ICERs per 5-year age-group over a lifetime were also calculated, providing an overview of which age groups are more cost-effective to vaccinate. From Figure 2, ICERs remain less than the commonly accepted threshold £30,000/QALY gained[31] for persons aged less than 85, with the lowest ICERs from a NHS perspective found for those aged 65 to 69 (£10,275 per QALY gained) and for those aged 60 to 64 (£ 10,984 per QALY gained). By summing the incremental costs and QALYs of the vaccinated populations of interest, the overall ICER associated with any age-specific vaccination strategy can be observed. For instance, vaccinating only those aged 50 to 59 results in ICERs of £12,539 and £9,043, vaccinating only those aged 60 to 69 results in ICERs of £10,639 and £9,742, and vaccinating all persons aged 65 and older results in ICERS of £14,385 and £14,294, from a NHS and societal perspectives respectively. As productivity costs are limited to those aged less than 70 in the model, the NHS and societal perspective costs become identical after this age threshold.

The cost-effectiveness of vaccinating single age groups with comparable population size (10,000 people) was also performed; however, as the model is structured to support input data only to the detail of 5-year age bands, the analysis of a single age group resulted in nearly identical ICERs as that of the 5-year age group it falls within. For example, if a vaccination strategy was applied to those aged 66 only (a population size of 557,469), the ICERs would be £10,179 and £9,938 for the NHS and societal perspectives, respectively. The ICER differs slightly from that of the aged 65 to 69 ICER due to the slight difference in the gender ratio of this smaller population as well as differential mortality rates, utility values, employment rates, and incidence of HZ.

### Sensitivity Analysis

**Figure 3:**
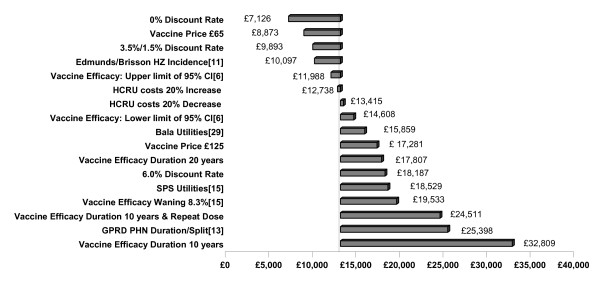
**Selected Deterministic Sensitivity Analysis: Tornado Diagram**.

Deterministic sensitivity analyses were performed to assess scenario uncertainty in the model. The tornado diagram in Figure 3 illustrates the difference from the base case in the lifetime cost per QALY observed for a number of selected sensitivity analyses performed from a NHS perspective. Societal perspective results are not presented, as the relative sensitivity of changes to this perspective is similar to those from a NHS perspective.

A number of analyses were performed to explore the uncertainty around the base case assumptions of vaccine efficacy, testing the applied point estimate and duration of vaccine efficacy. The lower and upper limit of the 95% confidence interval of the vaccine efficacy obtained from the Oxman trial were tested in the sensitivity analysis, indicating a moderate impact on the ICERs, *i.e*. an 8% increase and a 12% decrease respectively. In addition, the base case assumes that the efficacy of the vaccine is constant over the lifetime of the population, therefore an assumption of a limited duration of full vaccine efficacy (20 years and 10 years) followed by a lifetime of no efficacy was examined. As expected, the shorter durations of protection have an adverse impact on the expected benefits of vaccination, resulting in higher ICERs of £17,807/QALY and £32,809/QALY gained respectively. Another sensitivity analysis assumed a 10-year duration of full vaccine efficacy and, in addition, that 50% of the remaining healthy vaccinated population would receive a repeat dose after 10 years, which provided them with full lifetime efficacy. From the NHS perspective, the inclusion of a repeat dose results in an ICER of £24,511/QALY. A final sensitivity analysis applied an 8.3% annual waning rate, reported by Pellissier *et al*. as the upper limit of the waning rate [15], from the first month following vaccination. This resulted in an ICER of £19,533/QALY gained.

Discount rates of 0% and 6.0% for both costs and benefits, as well as a scenario of 3.5% for costs and 1.5% for benefits were tested. Results were quite sensitive to the values chosen. Lower discount rates will decrease ICERs to a large degree. Higher discount rates tend to emphasise vaccination costs, which are not affected by discounting as they are incurred in the first year, while diminishing the clinical benefits and cost savings achieved in subsequent years.

The vaccine coverage rate applied in the base case analysis was the same across all age groups, *i.e*. 40%. The use of differential coverage rates was explored, assuming that different age groups are covered by the vaccine at different rates, *i.e*. 60% of those aged 50-64, 30% of those aged 65-84, and 10% for those over 85+ years. Under this assumption, the resulting ICER decreased to £12,483.

The GPRD analysis provided information on HZ and PHN, covering the pain split as well as PHN duration [11]. A sensitivity analysis explored the effect of using GPRD data solely for all main HZ and PHN characteristics [11]. As can be seen in Table 1, the values are more conservative than the base case results. This is due primarily to the decreased number of moderate and severe pain cases of both HZ and PHN (compared to the SPS pain split).

Several sensitivity analyses were conducted to assess the impact of disease-specific utility values on results. Three sources for utility weights were available for mild, moderate, and severe pain associated with HZ and PHN. Alternative utility values, based on an HZ population, were available from Bala *et al*. [29] and the SPS [15]. For both of these alternative utility values, their associated decrements were lower than those reported by Oster [13]. The difference is mainly found in the severe pain state. Oster [13] reports a utility decrement from full health to HZ/PHN with severe pain of 0.75 whereas the Bala [29] and SPS [15] values imply that the decrement is 0.53 and 0.45, respectively. Because the decrements from the alternative studies are modest relative to the Oster [13] study, the total number of QALYs gained over the lifetime of vaccinated individuals is lower when using the Bala [29] and SPS [15] utility values, resulting in increased ICERs.

Several sensitivity analyses had only a marginal effect on results, *i.e*. the inclusion of HZ mortality [12], the assumption of no hospitalisation due to HZ/PHN, the increase of vaccine administration costs.

**Figure 4:**
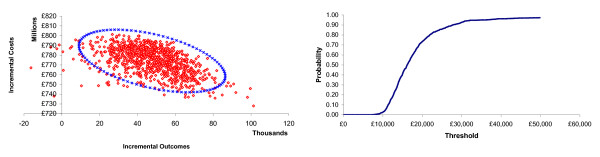
**Probabilistic Sensitivity Analysis - NHS Perspective: Scatter Plot of ICERs and Cost-Effectiveness Acceptability Curve**. Note: The points in the 4th quadrant of the scatter plot represent negative incremental outcomes, as during some of the PSA runs the vaccination policy will result in lower outcomes than the no vaccination policy.

Probabilistic sensitivity analyses were performed using Monte Carlo simulations (1,000 runs) on distributions for those parameters described earlier. Due to the inclusion of a new variable, the vaccine waning rate, and also since some means used for the sensitivity analysis differed from their corresponding base case value, the mean results of the PSA are not identical to the base case results. The mean result after 1,000 runs maintained the cost-effectiveness of the vaccination strategy, with a mean ICER of £19,551 (se: 58,922) for the NHS perspective. Figure 4A shows the NHS perspective for the outcome of QALYs gained. Presented in Figure 4B is the cost-effectiveness acceptability curve, providing the number of simulations which fall below given values or thresholds. The probability of not surpassing the commonly used threshold of £30,000/QALY from a NHS perspective is 92.7%. Therefore, the PSA illustrates the robustness of the cost-effectiveness of the vaccination policy.

## Discussion

The main results of this analysis, demonstrate numerous benefits of a vaccination strategy. These benefits include a reduced number of HZ and PHN cases, an increase in health-related quality of life as captured by the QALY, in addition to a reduction in hospitalisations, consultations and prescription costs for the vaccinated population. While sensitivity analyses illustrated that the model results are sensitive to some inputs, the vast majority of scenarios resulted in ICERs well below the cost-effectiveness thresholds commonly used in the UK [31].

This model takes into consideration a number of factors which contribute to its robustness. First, the model is population-based and therefore of direct relevance to decisions concerning vaccination, as this is done at the population-level. In addition, the model takes into account the ageing of the population over the duration of the model, adjusting for their mortality, the likelihood of contracting HZ and PHN and the efficacy of the vaccine. Though it was assumed that the vaccine would have lifetime duration of efficacy, the model is able to accommodate any changes to vaccine durability. Also this model incorporates epidemiological data that were obtained from a very large dataset (GPRD) [11], these input parameters for the model could be considered robust and representative of clinical practice in the UK.

Similar to other cost-effectiveness models, the main limitations of this study relate to the uncertainty surrounding some parameter estimates used in this model. One of these limitations concerns the assessment of disease severity in both HZ and PHN. The results of the severity split in HZ and PHN is quite different when the SPS and GPRD data are compared. For the base case we have opted for the SPS split for both HZ and PHN as we believe that this is the most accurate representation available from the literature, as special care was taken with regards to diagnosis in this study due to the use of the validated ZBPI questionnaire [16]. Assessment of severity in GPRD was based on treatments prescribed [11] rather than actual pain measured and therefore is expected to be less reliable than SPS data, where measurements were geared to providing clinical benefits. Experts further confirmed that the majority of patients experience pain at HZ onset which confirms the higher validity of the SPS data.

Utility weights were available from several sources [13,15,29] and Oster *et al*. [13] utilities, obtained in a PHN population, were selected for the base case due to the focus on neuropathic pain. A recent publication investigating the quality of life in neuropathic pain reported utility weights of 0.67 for mild, 0.46 for moderate and 0.16 for severe pain [25], indicating that the Oster values are appropriate for this study. In addition a recent publication by Van Hoek *et al*. [8] arrived at similar utility scores for the different pain states, *i.e*. 0.78, 0.61 and 0.27 for mild, moderate and severe pain respectively. In the standard approach in economic evaluations, the utility gain from prevention is the utility that would otherwise be lost due to illness. An extension of the standard utility model and analysis of prevention interventions is offered by the utility-in-anticipation concept introduced by Cohen and Henderson [32]. This concept acknowledges the fact that the utility resulting from preventive measures such as vaccination follows immediately after vaccination until the time when the outcomes was expected. Furthermore this utility will depend on the anxiety associated with both the perceived risk of infection and the perceived effectiveness of the vaccination in reducing that risk. This model does not include any such gains and therefore could underestimate the total utility gained from vaccination. Obviously, had this been included, this would have resulted in lower ICERs.

A 3.5% discount rate for both costs and benefits was used in the base case analysis as suggested by the current National Institute of Health and Clinical Excellence (NICE) guidelines. A lower discount rate of 1.5% for outcomes was selected in the sensitivity analysis to account for the long-lasting effects of the vaccine, resulting in lower ICERs of £9,893 per QALY gained for the third party payer perspective. This reflects the previous guidelines set by NICE, which recognised that differential discounting is appropriate in certain cases [33]. This is because vaccination programmes accrue their cost in the present but may not observe their benefits until the future. This can be seen in the case of the vaccine preventing HZ where those vaccinated may likely be in their early 50s and 60s, but the incidence of HZ increases with age, thus the benefit of the vaccine may not be observed until the medium- to long-term. Discounting health benefits created by vaccines with long-term effects at the regular discount rates can negatively affect their true benefit by underestimating the cost-effectiveness of the vaccine [27].

Modelling the appropriate duration of vaccine efficacy is also a significant issue. Extensive SA were conducted to assess the impact of different duration of protection and showed that the highest ICERs were obtained when assuming a 10-year duration of efficacy, or utilising a waning rate. Most of the sensitivity analyses were still below £30,000/QALY.

Another limitation relates to the available vaccine efficacy data. Firstly, as the SPS trial did not include patients aged 50 to 59, the model assumed that the efficacy values for those aged 60 to 69 would be relevant for this younger population. Secondly, the SPS trial reported a 22% reduction in pain for those developing HZ [6], which may consequently reduce the pain associated with PHN, but as this effect was difficult to incorporate into the structure of the model as such, it was not taken into account directly.

Most of the resource use data were also taken from GPRD [11]. Though we are confident that primary care resource use was accurately recorded, there is less certainty over the secondary care data, as there were only a few referrals or hospitalisations due to HZ or PHN. It is possible therefore that the estimated treatment costs were underestimated. The underestimation of costs does have the advantage that it does not favour the vaccine arm and therefore represents a conservative approach. A sensitivity analysis which varied all health care costs 20% above and below their base case value found that this had minimal impact on the ICERs. Furthermore, the model provides a conservative estimate of the value of herpes zoster vaccination. By not incorporating the common ocular and neurological complications (other than PHN) of HZ, including keratitis, iritis, retinal necrosis, [34] meningitis, encephalitis, and myelitis, [35] due to current lack of available data, the model may underestimate the potential health benefits and cost savings resulting from vaccination. Therefore future analyses, in the form of additional retrospective or prospective studies, would be of interest in order to model more accurately such potential disease pathways following herpes zoster and their impact on costs and quality of life.

With regards to a real-world vaccination strategy, it is worth nothing that the results presented in this analysis would only apply for the first years of a vaccination programme where a "catch-up" programme would be instituted for older patients. Following this period, a vaccination strategy would typically include younger cohorts (for instance, those aged 50-69) as other older adults will have already received the vaccine. As a result, in these later years, the cost-effectiveness of vaccination would improve, as illustrated in this study by the lower ICERs associated with relatively lower age groups.

A health economic evaluation of the new live attenuated vaccine against herpes zoster in England and Wales was recently published by Van Hoek *et al*. [8] The Van Hoek *et al*. model employs a different categorisation of HZ/PHN pain states by including a state of clinically relevant pain (CRP) to characterize both moderate and severe pain, while it assumes a limited duration of efficacy with the use of a waning rate,. In addition, there are differences in several input parameters such as HZ incidence rates, disease-specific utilities, and vaccine price applied. Even though a direct comparison of the base case results of the two analyses is not possible, the reported sensitivity analysis applying the maximum vaccine protection duration (100 years) for the cohort aged 65 in the Van Hoek *et al*. study (£5,660/QALY), produced almost identical results to our study (£5,583/QALY) when we set the vaccine price and population size equal.

In addition, a previous model by Edmunds *et al*. [12] had estimated the potential cost-effectiveness of vaccination prior to the availability of data on the clinical efficacy of such a vaccine. Structurally, this previous model differs from ours due to a different and less detailed utilisation of pain states and because it was not able to model the multiple efficacies (direct and indirect) now known of the vaccine. Despite these differences, as well as the use of several input parameters which relied on secondary data, considering a cohort aged 65, the base case resulted in ICERs of similar magnitude to our study, with values below £10,000 per QALY gained from a NHS perspective.

A third cost-effectiveness study by Pellissier *et al*. [15] has been published in the US, and while results cannot be directly compared to the UK due to differences in epidemiology and related health care costs, the general model structure has many similarities to the current model. Age-specific and severity-specific data were considered where possible. Vaccine efficacy was modelled using several dimensions, with both models allowing the use of a waning rate (however neither of the two studies included this feature in the base case analysis). In both studies, retrospective database analyses were performed to inform economic inputs and the resulting ICERs were determined to be cost-effective using locally accepted thresholds. Therefore, it confirms the robustness of the methods used in this analysis.

There are a number of areas where additional research could further improve the accuracy of the model. Continued validation of the duration of efficacy of the vaccine, as mentioned above, remains one of the key areas for research.

## Conclusions

The main results of this cost-effectiveness analysis show that a vaccination programme preventing HZ and PHN is likely to lead to substantial health and economic benefits for the UK. The model predicts that the most cost-effective strategies for the NHS are to vaccinate people between 60-64 and 65-69 years (£10,984 and £10,275). But beyond these age-groups, in most scenarios, the cost-effectiveness ratios remain below the commonly accepted cost-effectiveness threshold in the immunocompetent population aged 50 years and more.

## Competing interests

VR and MB are employed by SPMSD who funded the study. All other authors declare that they have no competing interests.

## Authors' contributions

MM developed the cost-effectiveness model and drafted the manuscript. LM programmed the cost-effectiveness model and drafted the manuscript. AM validated the cost-effectiveness model and contributed to the drafting of the manuscript. VR reviewed the design and results of the cost-effectiveness model and participated in the drafting of the manuscript. MB reviewed the design and results of the cost-effectiveness model and participated in the drafting of the manuscript. All authors read and approved the final manuscript.

## Supplementary Material

Additional file 1 **Vaccination Characteristics**. Supplemental data. Click here for file

Additional file 2 **Calculation of Utilities**. Supplemental data. Click here for file
