# Supplementary material for: A health economic model for evaluating a vaccine for the prevention of herpes zoster and post-herpetic neuralgia in the UK
Source: Cost Eff Resour Alloc. 2010 Apr 30;8:7. doi: 10.1186/1478-7547-8-7 (PMC2881001; doi:10.1186/1478-7547-8-7)
Supplement: Additional file 1 — Vaccination Characteristics. Supplemental data. [file 1478-7547-8-7-S1.DOC]

### Additional file 1. Vaccination characteristics

The model assumes that the vaccine has a direct and an indirect effect on the number of cases of PHN.

The vaccine has a direct effect on the number of PHN cases, which is more marked in the older age group. During the model validation process with the Oxman trial results, it was found that using the then reported PHN – direct vaccine efficacy value for ages 70+ of 47% (this value has since been updated to 49% [30]) produced a greater number of reductions in PHN cases for the model than was found in the Oxman trial [6]. This value was thus calibrated downwards to match the Oxman trial results, leading to a final PHN vaccine efficacy value of 30% for those aged 70+. This calibrated value of 30% is within the 95% confidence interval for PHN vaccine efficacy from the clinical trials thus providing further validation of the use of this value [36].

Adding the PHN direct and indirect effect results in the total effect as reported in the Oxman trial. The direct vaccine effect on PHN was included in the model, while the indirect effect was included implicitly as this was an additional effect of the direct HZ effect.

Table A1. Vaccine efficacy on HZ and PHN cases

| **Vaccine efficacy** | | | |
| --- | --- | --- | --- |
| **Age group** | **HZ - direct** | **PHN - direct** | **PHN - total** |
| 50-69 | 63.90% | 4.80% | 65.70% |
| 70+ | 37.60% | 30.00% | 66.80% |

*This value has been decreased from its original value of 47% during the validation process

Source: CDC/Recommendation ACIP, Oxman 2005[6] for ‘PHN-total’
