# Supplementary material for: A health economic model for evaluating a vaccine for the prevention of herpes zoster and post-herpetic neuralgia in the UK
Source: Cost Eff Resour Alloc. 2010 Apr 30;8:7. doi: 10.1186/1478-7547-8-7 (PMC2881001; doi:10.1186/1478-7547-8-7)
Supplement: Additional file 2 — Calculation of Utilities. Supplemental data. [file 1478-7547-8-7-S2.DOC]

### Additional file 2. Calculation of Utilities

The following utilities obtained from Oster [13] were used to derive pain decrements:

Table B1. Pain-specific utility values

| Herpes zoster | **Utility value** | **Annual Decrement** | **Monthly Decrement** |
| --- | --- | --- | --- |
| No pain | 1.00 | 0.00 | 0.00 |
| Mild | 0.69 | 0.31 | 0.03 |
| Moderate | 0.58 | 0.42 | 0.04 |
| Severe | 0.25 | 0.75 | 0.06 |
| Post-herpetic Neuralgia | **Utility value** | **Annual Decrement** | **Monthly Decrement** |
| Mild | 0.69 | 0.31 | 0.03 |
| Moderate | 0.58 | 0.42 | 0.04 |
| Severe | 0.25 | 0.75 | 0.06 |

Source: Oster [13]

In addition to utility decrements, as a relatively elderly population is being considered, age-specific utilities were used from the Health Survey for England [23]. Table B2 below shows the values used for the different age groups. The utilities obtained from the Health Survey for England are for 10-year age bands. 5-year age-specific utility values were obtained from the Canadian HUI Mark 3 [24] and utilised for extrapolation to the UK population.

Table B2. Age-specific utility values by gender

| **Age group** | **50 - 54** | **55 - 59** | **60 - 64** | **65 - 69** | **70 - 74** | **75 - 79** | **80 - 84** | **85+** |
| --- | --- | --- | --- | --- | --- | --- | --- | --- |
| **Males** | 0.842 | 0.798 | 0.802 | 0.816 | 0.784 | 0.793 | 0.727 | 0.588 |
| **Females** | 0.833 | 0.778 | 0.782 | 0.775 | 0.745 | 0.740 | 0.680 | 0.549 |

Source: Health Survey for England [23], HUI-3 [24]

Since non-gender related 5-year age groups in the UK population are evaluated at one time, overall utility values were calculated from this data by weighting the male and female utility values by their respective proportional contribution to their age class. For instance, since, according to UK National Statistics, males account for 49% and females 51% of the UK 50-54 age group, the overall age-specific utility value was calculated as (49% * 0.842) + (51% * 0.833) = 0.837.

The utilities related to the pain states are applied to the age-specific utilities in an additive manner: the utilities provided by Oster [13] are used as decrements which are then subtracted by the age-specific utilities. Thus, following the above example, a 50-54 year old who has severe pain will have a utility value of 0.837 - 0.75 = 0.087. Once it was confirmed that females were more likely to suffer from both HZ and, the absolute decrement values were further decreased by calculating the additional reduction in overall utilities for a population which consisted of proportionally more females with lower utility values, thus reflecting the true HZ and PHN populations.
